# A rare case of ocular IgG4-related disease in a pediatric patient

**DOI:** 10.11604/pamj.2025.51.89.48728

**Published:** 2025-08-07

**Authors:** Khadija Mouaddine, Bouchra Chkirate

**Affiliations:** 1 Department of Rheumatology, Nephrology, and Pediatric Cardiology, Rabat Children's Hospital, Rabat, Morocco,; 2 Faculty of Medicine and Pharmacy, Mohamed V University, Rabat, Morocco

**Keywords:** IgG4, ocular, pediatric, rituximab

## Image in medicine

Orbital IgG4 disease is a rare cause of bilateral exophthalmos. We report, through this observation, the first case of this disease in a child in our country. Our patient is a 13-year-old girl presenting with bilateral exophthalmos evolving for 4 months. The ophthalmic examination revealed preserved visual acuity with grade 1 papilledema. Orbital imaging showed the presence of a bilateral fusiform intraconical process encompassing the optic nerve and oculomotor muscles with grade III exophthalmos. There was no argument on imaging or bone marrow smear of a neoplastic origin. The urinary catecholamine dosage, immunological assessment, and angiotensin-converting enzyme (ACE) level were normal. An initial biopsy with immunohistochemistry (IHC) revealed reactive lymphocytic infiltration without signs of infection or tumor. The patient underwent intravenous steroid boluses, which rapidly improved her symptoms, then switched to oral steroids, but with multiple relapses during the regression. Surgical decompression was performed. A second biopsy revealed chronic fibrotic and inflammatory changes expressing IgG4, consistent with IgG4 disease. The IgG4 level returned to normal. The diagnosis of orbital hyper-IgG4 syndrome was made based on the histological findings. The patient received a corticosteroid bolus and rituximab, with good progress.

**Figure 1:**
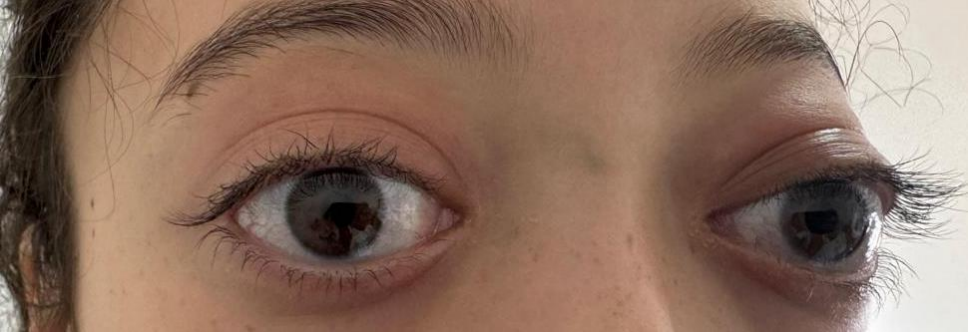
bilateral exophthalmos is more marked on the left in our patient

